# COVID-19 in WHO African Region: Account and Correlation of Epidemiological Indices with Some Selected Health-related Metrics

**DOI:** 10.4314/ejhs.v31i6.2

**Published:** 2021-11

**Authors:** Henshaw Uchechi Okoroiwu, Christopher Ogar Ogar, Dennis Akongfe Abunimye, Ifeyinwa Maryann Okafor, Ikenna Kingsley Uchendu

**Affiliations:** 1 Department of Medical Laboratory Science, Faculty of Basic Medical Sciences, Arthur Jarvis University, Akpabuyo, Cross River State, Nigeria; 2 Hematology Unit, Department of Medical Laboratory Science, Faculty of Allied Medical Sciences, University of Calabar, PMB 1115 Calabar, Nigeria; 3 Department of Medical Laboratory Science, University of Nigeria, Enugu Campus, 40001, Enugu, Nigeria

**Keywords:** COVID-19, SARS-Cov-2, COVID-19 in Africa, Coronavirus

## Abstract

**Background:**

The coronavirus disease 2019 (COVID-19) is a highly contagious and pathogenic viral disease caused by severe acute respiratory syndrome coronavirus-2 (SARS-CoV-2). Since it was first reported in Wuhan, China, it has spread across the continents. The study is aimed at describing epidemiological indices of COVID-19 as reported by the World Health Organization and to examine correlations with some country specific measures of general health status.

**Methods:**

Data from the WHO African region were extracted from World Health Organization, Global Health Security Index, Worldometer and World Bank databases, as at September 8, 2020. Other epidemiological indices were computed for the various countries. Epidemiological indices of COVID-19 were correlated with some selected health related metrics: Global Health Security index (GHSI) and current health expenditure (CHE). Pearson correlation was used to access the relationship between the health-related metrics and epidemiological indices.

**Results:**

Forty-seven (47) countries belonging to the WHO African region were evaluated. A total of 1,086,499 confirmed cases and 23,213 deaths were recorded giving a fatality rate of 2.1%. South Africa recorded the highest cumulative confirmed cases as well as deaths (Cases: 639,362; Deaths: 15,004) while Seychelles (Cases:135) and Eritrea/Seychelles (Deaths:0) had the least cumulative cases and deaths (135;0 and 330;0), respectively. South Africa recorded the highest attack rate (1127.67/100,000) while Republic of Tanzania recorded the least attack rate (0.78/100,000). The highest case fatality rate/ratio was observed in Chad (7.60%) while the least value was observed in Seychelles (0.0%). France was the most common country involved in travel history of index cases. Sporadic transmission was recorded in 3 countries, 9 countries had cluster of cases while the rest had community transmission. The first WHO African region country to record COVID-19 case was Algeria, while Comoros was the last. Significant positive correlation was found between COVID-19 case number/deaths and Global Health Security Index.

**Conclusion:**

The WHO African region has had its own share of the pandemic with all the countries being affected. The trio of cluster cases, sporadic and community transmission were recorded with majority being community transmission.

## Introduction

The Coronavirus disease 2019 (COVID-19) caused by the novel severe acute respiratory syndrome coronavirus 2 (SARS-CoV-2) has metamorphosed into global pandemic with noxious morbidity and mortality (1,2). The COVID-19 pandemic is the most pressing health care issue globally (3). At the onset of the pandemic it was broadly designated as a severe respiratory illness presenting with fever, atypical pneumonia, cough and dyspnea (4,5). However, altered sense of smell and taste have long been found to be associated with the disease (5,6). More so, a review of 77 observational studies showed a good number of patients presenting with less typical symptoms. (5,7).

As of July 22, 2021, SARS-CoV-2 has been responsible for 191,773,590 infections and 4,127,963 confirmed deaths (fatality rate: 2.15%) globally with the United States accounting for 33,875,385 cases and 604,546 confirmed deaths (the highest disease burden). The stratification of the data showed that the region of the Americas are currently the epicenter of the pandemic accounting for 39.22% (75,220,757) of the confirmed cases followed by Europe that accounted for 30.54% (58,576,440) of confirmed COVID-19 cases. Then in subsequent order South East Asia, Eastern Mediterranean, Africa and West Pacific regions (of WHO Classification) accounted for 19.39% (37,191,075), 6.26% (12,000,909), 2.44% (4,688,762) and 2.13% (4,094,883), respectively of global COVID-19 cases (8). Consequently, the WHO African region is the 5^th^ worst hit out of the six (6) WHO regions. This study is aimed at describing epidemiological indices of COVID-19 in WHO African region as reported by the World Health Organization and to examine correlations with some country specific measures of general health status.

## Methods

**Data collection**: Epidemiological indices of COVID-19 up to September 8, 2020 were collected from the official dashboard of World Health Organization (WHO) (9). The cumulative confirmed cases, cumulative deaths and attack rate per 100,000 of the population, transmission pattern and percentage of all deaths were extracted while case fatality ratio was computed for each country. The population of the various countries was extracted from Worldometer (10). While the health expenditure (%GDP) was extracted from World Bank database (11), the 2019 global health security index (GHSI) was extracted from the Global Health Security index database (12). Information on index cases were extracted from center for disease control/infectious disease control centers/COVID-19 update/COVID-19 information Hub/Ministry of Health web page of the various studied countries and WHO African region web page. The following search clauses were used: “World health organization African region+country index case”; “Country COVID-19 index case+country ministry of health”; “Country CDC”; “Country COVID-19 update dashboard”. In some cases, Africa news web page (13) was used to complement the details.

**Quality control on data extraction**: Data used in this study were extracted by the authors. The authors were divided into two groups: A (HUO and COO) and B (DAA and IMO). The two groups independently extracted the required data based on the study design. IKU harmonized the two independent results. In few cases of disparity all the authors double checked to ratify the correct data.

**The following operation definitions of terms/variables were used:**

**Global health security index:** Global health security index (GHSI) is the first comprehensive assessment and benchmarking of health security and related capabilities across the 195 countries that make up the state parties to International Health Regulations 2005. The index was intended to spur measurable changes in national health security and improve international capability to address world risk such as infectious diseases outbreak that could lead to pandemics. The GHS index relies entirely on open source information (data that the country has reported on its own or has been reported by an international entity). The index prioritizes both countries' capacities as well as existence of functional, tested, proven capabilities for stopping outbreaks source (12).

**Current health expenditure (%GDP)**: This refers to the level of current health expenditure expressed as a percentage of gross domestic product (GDP). Current health expenditure as a share of GDP provides an indication on the level of resources channeled to health relative to other uses. It shows the importance of health sector in the whole economy and indicates the societal priority which health is given measured in monetary terms. It is measured as percentage of GDP (%GDP) (14). It includes health care goods consumed each year and does not include capital health expenditure such as buildings, machinery (15).

**Confirmed cases/cumulative confirmed cases:** This refers to the total number of confirmed COVID-19 infection cases within the period of study. It is represented as frequency.

**Deaths/cumulative deaths**: This refers to the total number of deaths that resulted owing to COVID-19 infection within the study period. It is represented as frequency.

**Percentage of deaths**: This refers to the number of COVID-19 deaths recorded in a country in relation to the cumulative COVID-19 related deaths recorded in all the countries assessed within the study period. It is represented in percentage.

**Attack rate / Attack rate per 100,000 of population**: The index refers to the number of persons infected with COVID-19 per 100,000 of the country's population. It is represented as frequency per 100,000 of population.

**Case fatality rate/ratio**: It refers to the proportion of people who died from COVID-19 infection among all individuals diagnosed with COVID-19 over the studied period.

**Statistical analysis**: Retrieved data were analyzed with SPSS for windows, version 22 (IBM Corp Armonk, NY). Epidemiological and health related metrics were described using frequencies and proportions (percentages). Pearson correlation was used in determining association of some COVID-19 epidemiological indices with some health related cum financial metrics. The alpha value was benched at 0.05.

## Results

Forty-seven (47) countries belonging to the WHO African region were assessed. A total number of 1,086,499 cases and 23,213 deaths were recorded in the WHO African region giving a case fatality rate of 2.1% as of September 8, 2020. South Africa (639,362), Ethiopia (59,648) and Nigeria (55,160) were the leading countries in terms of cumulative confirmed cases, while Seychelles (135), Eritrea (330) and Mauritius (356) had the least cumulative confirmed cases. In terms of COVID-19 related deaths, South Africa (15,004), Algeria (1,562) and Nigeria (1,061) had the highest values while Seychelles (0), Eritrea (0) and Burundi (1) had the least cumulative deaths. South Africa (1,127.67), Cape Verde (788.99) and Equatorial Guinea (515.11) recorded the highest attack rates per 100,000 of the population, while Republic of Tanzania (0.79), Niger (3.24) and Burundi (3.54) had the least attack rates per 100,000 of population. The highest case fatality rates were observed in Chad (7.60%), Liberia (6.25%) and Niger (5.86%) while Seychelles (0), Eritrea (0) and Burundi (0.21) had the least case fatality rates. Eight (8) countries: Burkina Faso, Cameroon, Lesotho, Congo, South Africa, Mauritius, Togo and Rwanda were involved in transmission history of the index cases while sixteen non-African countries: Italy, Portugal, France, UK, United Arab Emirate (Dubai), Spain, Norway, USA, Japan, Turkey, Belgium, Switzerland, Saudi Arabia, India, Netherlands and Germany were involved in the transmission history of index cases (Table 1)

**Table 1:** Account and epidemiological indices of COVID-19 and some health-related metrics among the WHO African region countries up to September 8, 2020

Country	Confirmed cases	Deaths	% of all death	Attack rate	Case Fatality rate	Date of index case	Transmission pattern	Route of index case	Population	GHS-I	Health exp. (%GDP)
Algeria	46653	1562	6.73	108.46	3.35	Feb. 25	CT	Italy	43851044	23.6	6.37
Angola	2981	120	0.52	10.19	4.03	March 21	Clusters	Portugal	32866272	25.2	2.79
Benin	2213	40	0.17	17.72	1.81	March, 16	CT	Burkina Faso	12123200	28.8	3.72
Burkina Faso	1452	55	0.24	7.03	3.79	March, 9	CT	France	20903273	30.1	6.92
Botswana	2126	9	0.04	86.79	0.42	March 30	Clusters	UK, Thailand.	2351627	31.1	6.13
Burundi	466	1	0.00	3.54	0.21	March 31	Clusters	Rwanda, Dubai.	11890784	22.8	7.52
Cameroon	19848	415	1.79	71.18	2.09	March, 6	CT	France	26545863	34.4	4.67
Cape Verde	4358	42	0.18	788.99	0.96	March, 20	Clusters	UK	555987	29.3	5.17
Chad	1040	79	0.34	4.61	7.60	March, 19	CT	Cameroon	16425864	28.8	4.49
CAR	4729	62	0.27	22.81	1.31	March, 14	CT	Italy	4829767	27.3	5.82
Congo	4891	114	0.49	82.20	2.33	March, 14	CT	France	5518087	23.6	2.93
Comoros	448	7	0.03	50.73	1.56	April 30	CT	France	869601	27.2	7.38
Cote d'Ivoire	18701	119	0.51	73.05	0.64	March, 11	CT	Italy	26378274	35.5	4.45
DRC	10233	260	1.12	10.88	2.54	March, 10	CT	France	89561403	26.5	3.98
Equatorial Guinea	4985	83	0.36	515.11	1.66	March, 14	CT	Spain	1402985	16.2	3.11
Eritrea	330	0	0.00	5.56	0.00	March, 21	Sporadic	Norway	3546421	22.4	2.87
Eswatini	4884	94	0.40	359.36	1.92	March, 14	CT	USA, Lesotho	1160164	31.1	6.93
Ethiopia	59648	933	4.02	52.86	1.56	March, 13	CT	Japan	114963588	40.6	3.50
Gabon	8608	53	0.23	449.31	0.62	March, 12	CT	France	2225734	20.0	2.78
Gambia	3196	99	0.43	136.83	3.10	March, 17	CT	UK	2416668	25.2	3.28
Ghana	44777	283	1.22	146.63	0.63	March, 12	CT	Norway, Turkey	31072940	35.5	3.26
Guinea	9816	62	0.27	68.39	0.63	March, 13	CT	Belgium	13132796	32.7	4.12
Guinea Bissau	2245	38	0.16	105.97	1.69	March, 25	CT	Congo, India	1968001	20.0	7.24
Kenya	35205	599	2.58	67.29	1.70	March, 13	CT	USA	53771296	47.1	4.80
Liberia	1311	82	0.35	25.73	6.25	March, 16	CT	Switzerland	5057681	35.1	8.16
Lesotho	1164	31	0.13	51.71	2.66	May, 13	Clusters	South Africa, Saudi Arabia	2142249	30.2	8.76
Madagascar	15352	202	0.87	55.23	1.32	March, 20	CT	France, Mauritius,	27691018	40.1	5.50
Mali	2870	127	0.55	13.98	4.43	March, 25	CT	France	20250833	29.0	3.79
Mauritania	7164	160	0.69	157.58	2.23	March, 13	CT	Europe	4649658	27.5	4.40
Mauritius	356	10	0.04	27.57	2.81	March, 18	Sporadic	Belgium	1271768	34.9	5.72
Malawi	5621	176	0.76	28.11	3.13	April, 2	CT	India	19129952	28.0	9.65
Mozambique	4557	27	0.12	14.24	0.59	March, 22	CT	UK	31255435	28.1	4.94
Niger	1178	69	0.29	3.24	5.86	March, 19	Clusters	Togo	24206644	32.2	7.74
Nigeria	55160	1061	4.57	26.62	1.92	Feb. 27	CT	Italy	207151803	37.8	3.76
Namibia	8810	91	0.39	321.09	1.03	March, 14	CT	Spain	2540905	35.6	8.55
Rwanda	4409	19	0.08	33.61	0.43	March, 14	Clusters	India	12952218	34.2	6.57
Sao Tome & Principe	898	15	0.06	426.37	1.67	April, 6	Clusters	†	219157	17.7	6.23
Senegal	13987	290	1.23	80.14	2.07	March, 2	CT	France	16743927	37.9	4.13
Seychelles	135	0	0.00	136.52	0.00	March, 14	Sporadic	Italy	98347	31.9	5.01
Sierra Leone	2055	71	0.31	28.86	3.45	March 31	CT	France	7976983	38.2	13.42
South Africa	639,362	15004	64.64	1127.67	2.35	March, 5	CT	Italy	59308690	54.8	8.11
South Sudan	2552	49	0.21	17.2	1.92	April, 5	CT	Netherlands	11193725	21.7	9.76
Togo	1488	32	0.14	18.01	2.15	March, 6	CT	France, Benin Germany Turkey	8278724	32.5	6.20
Uganda	3776	44	0.19	7.7	1.17	March, 22	Clusters	Dubai	45741007	44.3	6.19
United Rep. of Tanzania	509	21	0.09	0.79	4.13	March, 16	CT	Belgium	59734218	36.4	3.65
Zambia	12836	295	1.27	67.85	2.3	March, 18	CT	France	18,383955	28.7	4.47
Zimbabwe	7116	208	0.89	40.78	2.92	March, 21	CT	UK	14862924	38.2	6.64

Afri: African, UK: United Kingdom, CT: Community transmission, CAR: Central African Republic, DRC: Democratic Republic of Congo, Rep: Republic; GDP: Gross domestic product, GHS-I: Global health security index, †: Country not disclosed. All dates refer to 2021

The detailed description of the index cases of the various WHO African Region countries studied is shown in Table 2.

**Table 2:** Description of the index cases of the various countries studied

Country	Description of index case	Country	Description of index case	Country	Description of index case
Algeria	An Italian who arrived the country on February 17, 2020 from Italy.	Eswatini	A 33 year-old female with travel history from USA at the end of February, 2020, then to Lesotho.	Niger	A 36 year-old Nigerian man. He had travelled to Lome, Accra, Abidjan and Ougadougou.
Angola	Two persons returning from Portugal on March 17–18, 2020. The 1^st^ case was a Sonangol employee who flew from Lisbon to Luanda. The 2^nd^ case had flown in from Porto.	Ethiopia	Japanese who travelled from Japan to Burkina Faso and arrived in Ethiopia.	Nigeria	An Italian man who flew from Milan Italy to Nigeria on February 25, 2020.
Benin	A 49 year- old Burkinabe citizen who entered Benin on March 12, 2020 from Belgium and Burkina Faso.	Gabon	A 27 year old Gabonese man who had recently entered Gabon from France 4 days prior to confirmation.	Namibia	The two cases were a Romanian couple who arrived in Windhoek from Spain via Doha, Qatar, on 11 March, 2020.
Burkina Faso	Husband and wife, had recently returned to Burkina Faso from a trip to France.	Gambia	A woman in her thirties who had travelled to the Gambia from the United Kingdom on 15^th^ March, 2020.	Rwanda	An Indian citizen who arrived from Mumbai, India on March 8^th^, 2020.
Botswana	2 males and 1 female who travelled to UK and Thailand.	Ghana	The two patients who tested positive for the virus had arrived from Norway and Turkey to Ghana.	Sao Tome & Principe	4 cases reported at once without disclosure of travel history
Burundi	A Burundian returning from Rwanda: A Burundian returning from Dubai via Rwanda.	Guinea	An employee of EU delegation to Guinea who travelled from Brussels in Belgium to Conakry, Guinea.	Senegal	A French national and a resident of Senegal who returned to Dakar from France on 26^th^ February, 2020.
Cameroon	A 58 year-old French national who arrived in 2020. Cameroon on February 24, 2020.	Guinea Bissau	The first two confirmed cases were: a Congolese U. N. employee and an Indian citizen.	Seychelles	The two cases were Seychellois who returned from Italy.
Cape Verde	A 62 year-old British national who arrived on the island of Boa Vista on March 9 and started to show symptoms of fever and cough on March, 16.	Kenya	A 27 year old Kenyan woman who returned from the USA via London, UK on the 5^th^ March, 2020.	Sierra Leone	A 37 year-old man who travelled from France to Sierra Leone on 16^th^ March, 2020.
Chad	A Moroccan national who had travelled to Chad from Cameroon.	Liberia	Liberian government official who returned from Switzerland to Liberia.	South Africa	A South African national who returned from Italy.
Central Afri. Rep	A 74 year-old Italian man who returned to Central African Republic from Milan, Italy.	Lesotho	A positive result from Lesotho citizen travelers among the 81 citizens/ travelers from South Africa and Saudi Arabia	South Sudan	A 29 year-old United Nations staff (woman) who returned from Netherlands via Addis Ababa on February 28^th^, Sierra Leone and resided there for five weeks.
Congo	A 50 year-old Franco-Congolese national who arrived in Congo on 1^st^ March, 2020 from Paris after a brief stay in Amsterdam, Holland.	Madagascar	3 cases: A 41 year-old Malagasy citizen returning from France via Air France on March 17, 2020; A 19 year-old Malagasy citizen returning from Mauritius via Air Mauritius on 18^th^ March, 2020; A 45 year-old Malagasy citizen returning from France via Air Madagascar on March 19^th^, 2020. All were women.	Togo	A 42 year-old female Togolese who recently travelled to France, Benin, Germany and Turkey but returned to Lome through Benin by road.
Comoros	A 50 year-old Franco-Comorian who has since been admitted since April 23, 2020. The patient came into contact with a national with recent travel history to France.	Mali	2 Malian nationals that returned from France on 12^th^ and 16^th^ March, 2020: A 9 year old woman and a 62 year old male.	Uganda	A 36 year-old Ugandan male who travelled to Dubai on 17^th^ March, 2020 and returned on 21^st^ March, 2020.
Cote d'Ivoire	A 45 year-old Ivorian man who went to the hospital with complaints of fever and a runny nose after returning from Italy.	Mauritania	The case is an expatriate from a yet to be disclosed country in Europe who arrived in the Mauritanian capital of Nouakchott on March 9, 2020.	United Rep. of Tanzania	A 46 year-old female Tanzanian who departed the country on 3^rd^ March 2020 to Belgium and had visited Denmark and Sweden between the dates 5 and 13 March 2020. On the 15^th^ March 2020, she flew back to Tanzania from Belgium.
Democratic Rep. of Congo	The patient is a Congolese citizen who returned from France.	Mauritius	3 cases: A 52 year-old man who had dual Belgian and Mauritian nationality that returned to Mauritius from Belgium on 21^st^ Feb. 2020; A 52 year old man with both British and Mauritius nationality who returned to Mauritius on March 7, 2020; A 21 year old male cruise ship male worker that entered Mauritius on March 14, 2020.	Zambia	The two cases were a couple that had travelled to France on holiday.
Equatorial Guinea	A 43 year-old woman in Malabo, who returned to Equatorial Guinea from Madrid Spain.	Malawi	The 3 cases include a Malawian of Asian origin who travelled back from India, her relative and their housemaid.	Zimbabwe	A 43 year-old tourist who travelled back the UK through South Africa to Zimbabwe.
Eritrea	An Eritrean national arriving Asmara in Eritrea from Norway.	Mozambique	A 75 year-old man who had recently returned from Britain midway through March, 2020.		

**Figure 1:**
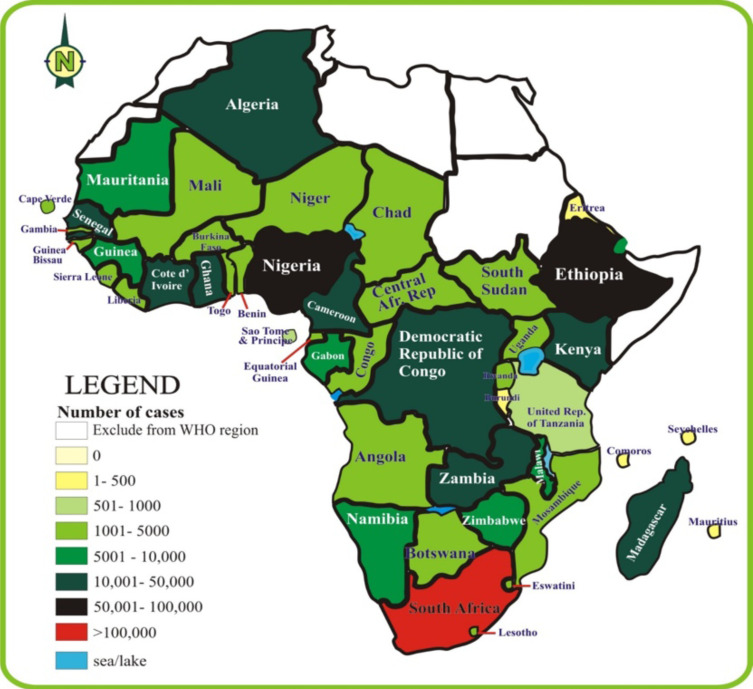
Diagrammatic representation of COVID-19 case distribution in the studied area.

Distribution of COVID-19 cases based on range in map of Africa indicating the WHO African regions are shown in Figure 1.

South Africa (639,362), Ethiopia (59,648) and Nigeria (55,160), were at the top of the list of countries with the top 10 confirmed COVID-19 cases while South Africa (15,004), Algeria (1,562), Nigeria (1,061), were at the top of the list of countries with top 10 COVID-19- related mortality. Analysis of the top 10 attack rates per 100,000 of the population showed South Africa (1,127.67), Cape Verde (788.99) and Equatorial Guinea (515.11) on top of the table. On the other hand, Chad (7.60%), Liberia (6.25%) and Niger (5.86%) were on the top of the list of the top 10 case fatality rates in the WHO African Region (Table 3).

**Table 3:** Summary of top 10 countries in the various COVID-19 epidemiological indices

COVID-19 epidemiological indices								

Rank	Confirmed cases		Deaths (% of all deaths)		Case fatality rate		Attack rate	
1^st^	South Africa	639,362	South Africa	15,004(64.64)	Chad	7.60	South Africa	1,127.67
2^nd^	Ethiopia	59,648	Algeria	1,562 (6.73)	Liberia	6.25	Cape Verde	788.99
3^rd^	Nigeria	55,160	Nigeria	1,061 (4.57)	Niger	5.86	Equatorial Guinea	515.11
4^th^	Algeria	46,653	Ethiopia	933 (4.02)	Mali	4.43	Gabon	449.31
5^th^	Ghana	44,777	Kenya	599 (2.58)	United Rep. of Tanzania	4.13	Sao Tome & Principe	426.37
6^th^	Kenya	35,205	Cameroon	415 (1.79)	Angola	4.03	Eswatini	359.36
7^th^	Cameroon	19,848	Zambia	295 (1.27)	Burkina Faso	3.79	Namibia	321.09
8^th^	Madagascar	15,352	Senegal	290 (1.23)	Sierra Leone	3.45	Ghana	146.63
9^th^	Senegal	13,987	Ghana	283 (1.22)	Algeria	3.35	Gambia	136.83
10^th^	Zambia	12,836	Dem. Rep. Congo	260(1.12)	Malawi	3.13	Seychelles	136.52

France was the most common country involved in the history of the transmission of the index cases in the studied countries involving 11 countries: Burkina Faso, Cameroon, Congo, Comoros, Democratic Republic of Congo, Gabon, Madagascar, Mali, Senegal, Zambia and Togo. On the other hand, Italy was involved in the history of the index case of 6 countries (Algeria, Cote d'Ivoire, South Africa, Seychelles, Central African Republic and Nigeria), while United Kingdom (UK) were involved in the transmission history of index cases of 5 countries (Botswana, Cape Verde, Gambia, Zimbabwe and Mozambique). Other countries noted were involved in less than 4 country index case history (Table 4).

**Table 4:** Stratification of various African countries and the corresponding country of origin of index case

Country of origin of index case	No. of countries involved	Countries affected	Route classification
Italy	6	Algeria, Cote d'Ivoire, South Africa, Seychelles, Central African Republic, Nigeria.	Intercontinental
Portugal	1	Angola	Intercontinental
Burkina Faso	1	Benin	Intracontinental
France	11	Burkina Faso, Cameroon, Congo, Comoros, Democratic Republic of Congo, Gabon, Madagascar, Mali, Senegal, Zambia, Togo*	Intercontinental
UK	5	Botswana, Cape Verde, Gambia, Zimbabwe, Mozambique	Intercontinental
Dubai	2	Burundi, Uganda	Intercontinental
Cameroon	1	Chad	Intracontinental
Spain	2	Equatorial Guinea, Namibia	Intercontinental
Norway	2	Eritrea, Ghana	Intercontinental
USA	2	Eswatini*, Kenya	Intercontinental
Lesotho	1	Eswatini*	Intracontinental
Japan	1	Ethiopia	Intercontinental
Turkey	1	Togo*	Intercontinental
Belgium	3	Guinea, Mauritius, United Republic of Tanzania	Intercontinental
Congo	1	Guinea Bissau	Intracontinental
Switzerland	1	Liberia	Intercontinental
South Africa	1	Lesotho*	Intracontinental
Saudi Arabia	1	Lesotho*	Intercontinental
Mauritius	1	Madagascar	Intracontinental
India	2	Malawi, Rwanda	Intercontinental
Togo	1	Niger	Intracontinental
Netherlands	1	South Sudan	Intercontinental
Germany	1	Togo	Intercontinental
Rwanda	1	Burundi	Intracontinental

* More than 1 travel history

Sporadic transmission was recorded in Eritrea, Mauritius and Seychelles while cluster of cases were observed in Angola, Botswana, Burundi, Cape Verde, Lesotho, Niger, Rwanda, Sao Tome and Principe, and Uganda. The remaining countries had community transmission on-going (Table 1).

**Figure 2:**
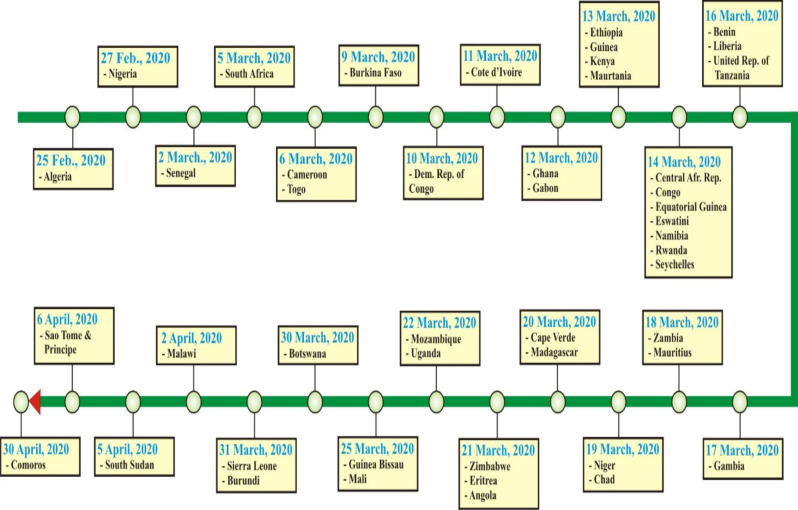
Chronological order of COVID-19 index case record of the various countries in WHO African region.

The first two African countries to record COVID-19 cases were Algeria (February 25, 2020) and Nigeria (February 27, 2020). These were followed by Senegal, South Africa, Cameroon, Togo, Burkina Faso, Democratic Republic of Congo, Cote d'Ivoire, Ghana, Gabon, Ethiopia, Guinea, Kenya, Mauritania, Central Africa Republic, Congo, Equatorial Guinea, Eswatini, Namibia, Rwanda, Seychelles, Benin, Liberia, United Republic of Tanzania, Gambia, Zambia, Mauritius, Niger, Chad, Cape Verde, Madagascar, Zimbabwe, Eritrea, Angola, Mozambique, Uganda, Guinea Bissau, Mali, Botswana, Sierra Leone and Burundi that recorded their index cases between March 2^nd^ and March 31^st^, 2020. The last 3 countries to record their index cases were South Sudan, Sao Tome and Principe and Comoros that had their index cases on 5^th^, 6^th^ and 30^th^ April, 2020, respectively (Figure 2).

Significant positive correlation (P<0.05) was observed between Global Health Security Index and cumulative confirmed COVID-19 cases, as well as cumulative COVID-19 related deaths and Global Health Security Index. Although non-significant correlation was recorded in cumulative confirmed COVID-19 cases vs Health expenditure; Cumulative deaths vs Health expenditure; Cumulative fatality rate vs Global Health Security Index; Cumulative fatality rate vs Health expenditure; Attack rate vs Global Health Security Index; Attack rate vs Health expenditure, their Pearson correlation coefficient are 0.120, 0.142, 0.035, 0.113, 0.177 and 0.024 (Table 5).

**Table 5:** Correlation results of some epidemiological indices and health service indicators

Correlated variables	Pearson correlation coefficient	P-value
Cumulative confirmed cases Vs GHSI	0.519	<0.01
Confirmed cases Vs Health Expenditure	0.120	0.422
Cumulative deaths Vs GHSI	0.495	<0.01
Cumulative deaths Vs Health Expenditure	0.142	0.342
Cumulative fatality rate Vs GHSI	0.035	0.816
Cumulative fatality rate Vs Health Expenditure	0.113	0.450
Attack rate Vs GHSI	0.177	0.239
Attack rate Vs Health Expenditure	0.024	0.873

Note: GHSI: Global health security index, Vs: versus

## Discussion

A total of 1,086,499 and 23,213 deaths were recorded in the WHO African region studied, giving rise to case fatality ratio of 2.1%. This placed the WHO African region 5/6 of the other WHO regions in terms of COVID-19 cases. The case fatality ratio observed in this study is below the global average (3.4%) (16) and that of the region of the Americas, Europe and South East Asia. In contrast to the Ebola Virus disease (EVD), the CFR is far below that of EVD which had global average of 50% (even up to 90% in some places) (17) and ranged from 39.6% to 84.3% in the West African Sub-region (20). However, COVID-19 has been found to be more contagious with an average Ro value of 3, consequently cause for higher cumulative number of deaths observed (19). Since the report of the first case in the region in Algeria on February 25, 2020, seven months later the epidemic curve in the region remained flatter in comparison with region of Americas, Europe and Asia as against earlier insinuations. Before now, African has been predicted to be most vulnerable continent in terms of COVID-19 infection and was predicted as region where COVID-19 will have major impact. This prediction was based on the continent's weak health care system cum large immunocompromised population (20,21,22). However, the prediction proved otherwise. There have been varying hypothesis in attempt to explain the reason to the relatively low COVID-19 cases in African region as against expectations. Some experts attributed this to low numbers of SARS-CoV-2 introduced (seeding) into Africa possibly due to low volume of air travel to the region (23). More so, the mitigative measures (partial and complete lock down and travel restrictions) (2) may have played role. Some researchers have proposed that the greater youthful population of the African region with median age < 20 years as against Europe and the USA with medium age > 38 years (24,25) is a contributing factor. Also, some authors have attributed the low incidence to favorable climate. Sajadi and colleagues have recorded association between temperature / humidity and COVID-19 spread (26) which is in line with previously reported factors for survival in earlier epidemic: SARS-CoV and influenza (27,28,29). Africa experiences warmer and drier weather within December and April season with average temperature of the day > 20 degree Celsius (30). In a different perspective, some authors have posited that a population across Africa have some level of SARS-CoV-2 immunity as a result of prior exposure to other coronaviruses (30). Lastly, there are postulations of prospective effect of Bacilli Calmette-Guerin (BCG) vaccination against COVID-19 infection (31). However, the reports were not from clinical trials (but experimental studies) which prompted WHO to recommend disregarding the results until the clinical trials are complete (32). Alternatively, there is possibility of low report of COVID-19 cases in African region owing to lack of material resources as available in the Americas and Europe continents that are more economically buoyant. Although some of the postulations have conflicting versions, future studies would help unravel the explicit contributing factors to the relatively low COVID-19 event in African region.

In this study, we found South Africa to be the epicenter of COVID-19 pandemic in the WHO African region. As of September 8, 2020, South Africa had 639,362 confirmed COVID-19 cases, accounting for more than half (58.8%) of the pandemic in the region. Comparatively to the global data, South Africa ranked 12^th^ in the global burden of COVID-19 pandemic below USA, India, Brazil, Russia, Argentina, Colombia, Spain, Peru, Mexico, France and UK. Though we could not “pin – point” the exact reason for such large burden, however, some authors have argued that South Africa carries a significant burden of tuberculosis, HIV, and HIV/TB coinfection, with millions of the population on immunosuppressant drugs as well as others who are HIV-positive but not on retroviral therapy (33). South Africa still has the largest global burden of HIV (approximately 19%) (34,35). There are reports that those with comorbidities are more susceptible to developing severe COVID-19 (36). On the flip side, some authors have attributed the “sky rocketing” of the pandemic in South Africa to arrival of winter in South Africa at the start of the epidemic considering the fact that all respiratory viruses spread more effectively in winter (20).

Adjusting the COVID-19 cases per 100,000 of the population (attack rate per 100,000 of population) still showed South Africa, then Cape Verde and Equatorial Guinea at the top. South Africa alone clocked attack rate above 1000 cases per 100,000 of the population. Although Cape Verde and Equatorial Guinea do not rank among the top 10 in terms of cumulative confirmed cases, cases per 100,000 of the population were observed to be high.

South Africa accounted for majority (64.64%) of all deaths in the region followed by Algeria and Nigeria. These three were among the first four countries in the region recording COVID-19 cases on March 5, February 25 and 27, respectively. As of the time of this report, Eritrea and Seychelles were the only countries in the region without record of COVID-19 related mortality.

Chad (7.60%), Liberia (6.25%) and Niger (5.86%) had the highest case fatality ratios. These values are quite far from the average CFR of the entire region and only comparable to values seen in countries in region of America and Europe such as Mexico (10.1%) and Italy (8.2%) (37). Although merely passed 1000 case profile, the case fatality rate of Chad is high and is among the highest values globally. Before the pandemic, Chad has been ravaged by malaria, Chikunguya and measles. The high incidence of measles have been attributed to insufficient vaccination (only 22% of children aged 12 to 23 months are vaccinated) (38). Inter-country comparison of CFR is an important indicator of disease characteristics and is important for both national and international priority settings as well as recognizing health system performance. However, it is pertinent to note that there are varying factors that can confound the value: undetected cases / low case detection, and delayed case reporting (39).

France was the most involved country in travel history of index cases in the region, followed by Italy and UK. A striking feature about countries that had index case with France travel history is that all except Democratic Republic of Congo and Zambia were all former French colonies. France still maintains very strong ties and less travel/visa bureaucracy with its former colonies more than their British (UK) counterpart (40).

In this study we found strong positive correlation between cumulative confirmed cases / cumulative deaths and Global Health Security Index. This observation is quite ironic in the sense that cases and deaths were supposed to be low in countries that have high GHSI and vice versa (negative correlation). This trend is also observed in global data of COVID-19 where countries with high GHSI such as USA and UK have high cumulative confirmed cases and deaths. Though we were not able to “pin point” the exact reason for this reverse trend, however, it can be inferred that countries with low human development index are less inclined to report or to put effort to get proper measures of COVID-19 cases and deaths. Nevertheless, future research and events would possibly substantiate this.

The findings of this study are potentially prone to varying limitations. First, the study took results in retrospect and the inherent limitations such as selection bias may not be ruled out. Secondly, the outcome at the time of going to press is not conclusive as the pandemic is still ongoing. Also, metric such as Global health security index (GHSI) relies on open-source information, it might be prone to bias.

In conclusion, the WHO African region has had its own share of the pandemic with all the countries being affected. The trio of cluster cases, sporadic and community transmission were recorded with majority being community transmission. Global health security index was found to be positively correlated with cumulative confirmed cases and cumulative deaths.
